# Clinical observation of different minimally invasive surgeries for the treatment of impacted upper ureteral calculi

**DOI:** 10.12669/pjms.296.3910

**Published:** 2013

**Authors:** Yuanhua Liu, Zhangyan Zhou, An Xia, Haitao Dai, Linjie Guo, Jiang Zheng

**Affiliations:** 1 Yuanhua Liu, Department of Urinary Surgery, First Affiliated Hospital of Yangtze University, Jingzhou 434000, P. R. China.; 2 Zhangyan Zhou, Department of Urinary Surgery, First Affiliated Hospital of Yangtze University, Jingzhou 434000, P. R. China.; 3 An Xia, Department of Urinary Surgery, First Affiliated Hospital of Yangtze University, Jingzhou 434000, P. R. China.; 4 Haitao Dai, Department of Urinary Surgery, First Affiliated Hospital of Yangtze University, Jingzhou 434000, P. R. China.; 5 Linjie Guo, Department of Urinary Surgery, First Affiliated Hospital of Yangtze University, Jingzhou 434000, P. R. China.; 6 Jiang Zheng, Department of Urinary Surgery, First Affiliated Hospital of Yangtze University, Jingzhou 434000, P. R. China.

**Keywords:** Transurethral ureteroscopic lithotripsy, Minimally invasive percutaneous nephrolithotomy, Retroperitoneal laparoscopic ureterolithotomy, Impacted upper ureteral calculus

## Abstract

***Objective: ***To compare the clinical effects of three minimally invasive surgeries on the treatment of impacted upper ureteral calculi.

***Methods:*** 135 patients with impacted upper ureteral calculi were selected and randomly divided into three groups (Group A-C) (n=45), which were treated with transurethral ureteroscopic lithotripsy, minimally invasive percutaneous nephrolithotomy, and retroperitoneal laparoscopic ureterolithotomy respectively. Relevant results of the three groups were compared.

***Results: ***The surgery time of Group C was significantly longer than those of Group A and Group B (P < 0.05). The postoperative hospitalization time of Group B was significantly longer than those of Group A and Group C (P < 0.05). 37.78% (17/45) of Group A patients required extracorporeal shock wave lithotripsy, being significantly more than those in Group B (6.67%, 3/45) and Group C (0, 0/45) (P < 0.05). The postoperative calculus clearance rate of Group A (51.11%, 82.22%) was significantly lower than those of Group B (91.11%, 97.78%) and Group C (93.33%, 100%) (P < 0.05). The incidence rates of postoperative complications in Group A-C were 11.11% (5/45), 8.89% (4/45) and 6.67% (3/45) respectively without significant differences (P > 0.05).

***Conclusion:*** The three surgical methods for impacted upper ureteral calculi should be selected according to practical conditions to improve therapeutic effects and to ensure safe surgery.

## INTRODUCTION

With continuous development of China's economy and society, great changes have taken place in Chinese people’s diet and lifestyle, which is accompanied by increasing incidence of lithiasis in urinary system, thus rendering it to be a common and frequently-occurring disease in clinic.^1^ Ureteral calculus is one of the main types of urinary calculi, with renal colic and hematuria as the main characteristics clinically, and is seriously endangering the life and work of patients. In particular, in case of impacted ureteral calculi, urination is rather difficult due to ureteral obstruction, which may lead to hydronephrosis.^2^ If timely intervention is not possible to relieve urinary obstruction, the patient’s renal function of the affected side may undergo progressive decline with extending course of hydronephrosis, posing a serious threat to the health of patients.^3^ As minimally-invasive surgical techniques continue to develop, there are a variety of methods for impacted upper ureteral calculi, which have achieved relatively satisfactory clinical efficacy.

This study compared the effects of three minimally-invasive surgical methods on the treatment of impacted upper ureteral calculi performed in our hospital, aiming to provide a reference for the clinical development of therapeutic strategies.

## METHODS

***General*** ***I*** ***nformation: ***A total of 135 patients with impacted upper ureteral calculi who were admitted to the Urology Surgery Department of our hospital from January 2011 to January 2013 were selected. The patients were divided into Group A, B and C according to a random number table (n=45). The patients were included according to the relevant standards of impacted upper ureteral calculi in the "Guidelines of urolithiasis diagnosis and treatment in China"^4^ formulated by the Lithology Group of Chinese Urological Association. In Group A, there were 25 males and 20 females, aged 26-54 years old, with the mean age of (43.41 ± 10.17) years old. In group B, there were 23 males and 22 females, aged 32-58 years old, with the mean age of (46.35 ± 10.31) years old. In group C, there were 26 males and 19 females, aged 23-53 years old, with the mean age of (44.73 ± 10.56) years old. The differences were not statistically significant in mean age, gender composition, state of illness and course of disease among the patients in the three groups (P>0.05) (Table-I).

***Evaluation*** ***C*** ***riteria: ***The patients were diagnosed according to the relevant standards of impacted upper ureteral calculi in the "Guidelines of urolithiasis diagnosis and treatment in China"^4^ formulated by the Lithology Group of Chinese Urological Association. All patients in this study showed varying degrees of recurrent history of pars lumbalis pain, and had incidental symptoms such as fever, hematuria, etc. After admission, the patients were examined by best color Doppler ultrasound scanning for urinary system, and auxiliary tests such as plain abdominal radiograph and excretory urography. They were then diagnosed as ureteral calculi. Meanwhile, according to the medical history of the patients, it was determined that the calculi had remained in ureter for over two months. Impacted upper ureteral calculi were finally confirmed by comprehensive diagnosis.

***Surgical*** ***P*** ***rotocols:*** The patients of the three groups were subjected to surgical treatments after chest radiograph, electrocardiogram, blood coagulation function and other auxiliary examinations that excluded surgical contraindications. The surgical protocols for the three groups were described as follows.

***Surgical protocol for Group A: ***The surgical protocol for Group A was transurethral ureteroscopic lithotripsy, which was performed under combined spinal-epidural anesthesia. The patient was in lithotomy position. Using zebra urological guidewire, rigid ureteroscope was entered into the ureter on the affected side through urethra to arrive at ureteral calculi to determine their size and location. Then the calculus was broken into small pieces by holmium laser, which were sucked out of human body by negative pressure of flowing water, and larger calculi were removed by lithotomy forceps. If calculi were enfolded by polyps, polypectomy was performed at first. After double-checking the elimination of calculi by ureteroscopy, double J stent was detained in ureter for drainage. Abdominal plain film examination was conducted 3 days after the surgery to evaluate its efficacy. In case of calculi larger than 4 mm in the kidney, extracorporeal shock wave lithotripsy was performed after the surgery for further treatment. The double J stent was removed about half a month after the surgery.

***Surgical protocol for Group B: ***The surgical protocol for Group B was minimally-invasive percutaneous nephrolithotomy, which was performed under general anesthesia with endotracheal intubation. The patient was in lateral position on the affected side using B ultrasound for assisted targeting. The kidney was punctured within the region formed by ribs 11-12, subscapularis linea and posterior axillary line, and middle renal calices were punctured conventionally. Then the percutaneous renal channel was gradually expanded, through which peelable thin sheath was placed and transferred to the rigid ureteroscope after complete expansion. With the renal calices entering into the ureter and reaching the calculi, if there were any in the renal calices, they were broken by holmium laser and removed out of human body. After being fixed by expansion sheath, calculi were broken into small pieces by holmium laser which were sucked out with the negative pressure of flowing water, and larger calculi were removed by lithotomy forceps. After calculus clearance, double J stent and nephrostomy tube were detained.

***Surgical protocol for Group C: ***The surgical protocol for Group C was retroperitoneal laparoscopic ureterolithotomy, which was performed under general anesthesia with endotracheal intubation. The patient was in lateral position on the uninjured side with the waist bridge elevated. A transverse incision of about 2 cm was cut at about 2 cm above the intersection of the ligature of bilateral iliac crests’ intersection and the intersection of mid-axillary line on the affected side. The subcutaneous tissue below the incision was bluntly dissected into the thoracolumbar fascia, entering into the retroperitoneal space through the thoracolumbar fascia and pushing the peritoneum aside with fingers through the retroperitoneal space to place airbag which was filled with 0.5 L of gas to expand the retroperitoneal space. Thereafter the airbag was taken out. Under the guidance of finger, Trocar was transferred to the site about 2 cm above the posterior axillary line iliac crest and that about 2 cm above the anterior superior iliac spine respectively through the retroperitoneal space. After the laparoscope and instruments for surgical procedures were placed inside, the airbag was filled with carbon dioxide, maintaining the control pressure at about 12-14mmHg. The renal fascia was cut open along the outer edge of quadratus lumborum, and the ureter was separated and freed from the perirenal fat and the medial plane of psoas muscle to find ureteral calculi. Both ureter and calculi were fixed, and then 2/3 of the ureteral wall above the calculi was cut open with electric knife to remove them. After the calculi were cleared, double J stent was detained in the ureter, the ureter was stitched, the retroperitoneal drainage tube was detained, and finally the surgical incision was sutured.

***Observation*** ***I*** ***ndices: ***The intra-operative and postoperative related indicators, postoperative complications and postoperative extracorporeal shock wave lithotripsy results of the three groups were compared. Plain abdominal radiograph was performed three days and one month after surgery respectively to determine further treatment methods. If the remaining calculi were less than 4 mm, the surgical removal of calculi was regarded successful. If not, the patients needed to receive extracorporeal shock wave lithotripsy subsequently.

***Statistical*** ***A*** ***nalysis: ***All the collected data were analyzed by SPSS 17.0. The measurement data were expressed as mean ± standard derivation ( ) and compared by t test. The numeration data were compared by Chi-square test. P<0.05 was considered statistically significant.

## RESULTS

***Comparison*** ***b*** ***etween Intraoperative*** ***a*** ***nd Postoperative Indices: ***The surgery time of Group C was significantly longer than those of Group A and B, and the time of Group B was significantly longer than that of Group A (P<0.05). The postoperative hospitalization time of Group B was significantly longer than those of Group A and Group C, and the time of Group C was significantly shorter than that of Group A (P<0.05). 37.78% (17/45) of Group A patients required extracorporeal shock wave lithotripsy, which was significantly more than those in Group B (6.67%, 3/45) and Group C (0, 0/45) (P<0.05). The difference was not statistically significant between the numbers of patients who received postoperative shock wave lithotripsy in Group B and C (P>0.05). The postoperative calculus clearance rate of Group A (51.11%, 82.22%) was significantly lower than those of Group B (91.11%, 97.78%) and Group C (93.33%, 100%) (P<0.05). No statistically significant difference was found between Group B and C in this rate (P>0.05) (Table-II).

**Table-I:** General information of the three groups

*Item*	*Group A (n=45)*	*Group B (n=45)*	*Group C (n=45)*
Disease course (month)	4.36±1.74	4.61±1.58	5.19±1.33
Calculus position (left/right)	23/22	24/21	23/22
Calculus volume (mm^3^)	148.13±27.52	146.85±30.36	149.16±32.15
Hydronephrosis degree (moderate/severe)	29/16	28/17	29/16

**Table-II:** Comparison between intraoperative and postoperative indices

*Item*	*Group A (n=45)*	*Group B (n=45)*	*Group C (n=45)*
Surgery time (min)	60.14±18.72	53.82±19.18^a^	87.92±18.37^ab^
Postoperative hospitalization time (d)	5.18±0.68	6.76±3.08^a^	4.55±0.48^ab^
No. of postoperative extracorporeal shock wave lithotripsy [n (%)]	17 (37.78)	3 (6.67)^a^	0 (0.00)^ab^
Calculus clearance rate on postoperative 3rd day [n (%)]	23 (51.11)	41 (91.11)^a^	42 (93.33)^a^
Calculus clearance rate on postoperative 1 month [n (%)]	37 (82.22)	44 (97.78)^a^	45 (100.00)^a^

Compared with Group A, ^a^P<0.05; compared with Group B, ^b^P<0.05

**Table-III:** Complications of the three groups [n (%)].

*Group*	*Fever*	*Ureteral perforation*	*Secondary ureterostenosis*	*Overall incidence rate*
Group A	2 (4.44)	1 (2.22)	2 (4.44)	5 (11.11)
Group B	3 (6.67)	0 (0.00)	1 (2.22)	4 (8.89)
Group C	3 (6.67)	0 (0.00)	0 (0.00)	3 (6.67)

***Postoperative*** ***C*** ***omplications: ***The incidence rates of postoperative complications in Group A, Group B and Group C were 11.11% (5/45), 8.89% (4/45) and 6.67% (3/45) respectively, without significant differences (P>0.05) (Table-III).

## DISCUSSION

We selected 135 cases of impacted upper ureteral calculi for three different treatments with pros and cons. Long surgeries are bound to jeopardize the patients in poor conditions. Calculus clearance rate and postoperative complications reflect therapeutic effects, while long hospitalization burdens patients economically.

Although retroperitoneal laparoscopic ureterolithotomy takes a long time, it is most prone to success. Moreover, postoperative extracorporeal shock wave lithotripsy can be avoided. Similarly, Liu et al.^5^ reported that only 6.67% of patients succumbed to fever, without severe complications such as ureteral perforation. However, this surgical technique is so sophisticated that only the surgeons adept at local anatomy are eligible.^6^

Minimally-invasive percutaneous nephrolithotomy is also effective. In this study, the clearance rate of Group B was similar to that of Group C. Nevertheless, this approach may induce large surgical trauma and significant bleeding, which is disadvantageous for the postoperative rehabilitation of patients.^7^ By considering the physical conditions of patients simultaneously, the method is more suitable for clearing large calculi complicated by renal calculi and severe hydronephrosis.^8^ In the meantime, this method is disadvantageous for the postoperative rehabilitation of patients, thus prolonging hospitalization that brings overwhelming economic burden.

In comparison, transurethral ureteroscopy lithotripsy does not function as effectively as the other two methods. Given that upper ureter is relatively wide, this surgical method is prone to inducing calculi ascending into the kidney by breaking them upward, thus hindering the utter clearance of calculi. In this study, Group A had the lowest rate of calculus clearance and the highest rate of postoperative extracorporeal shock wave lithotripsy, which are basically consistent with the results of Lai et al.^9^ This method avoids puncture trauma, reduces surgical bleeding and benefits postoperative rehabilitation, but about 11.1% of patients may suffer from postoperative complications.

The three methods exert different effects because urinary calculi form when urine is concentrated and precipitated as lumpy or granular materials that stay in the urinary system. Ureteral calculus, as a common type of urinary calculus, has been experiencing elevated incidence rate recently.^10^ The calculus, which originates from the descending of renal calculus into ureter, may give rise to incarceration and urinary obstruction with ureter narrowing, thereby leading to severe pain and hydronephrosis that exert serious influences on human health.^11^^,^^12^ Impacted ureteral calculi burden treatment by scratching the ureteral wall that forms enfolding ureteral polyps.^13^^,^^14^ In reference to this disease, conservative treatment suffers from low success rate and unstable clinical efficacy.^15^ Although extracorporeal shock wave lithotripsy may break some calculi, it fails to treat those enfolded by ureteral polyps.^16^ With continuous development of modern medicine, minimally-invasive surgery has been preferred in treating ureteral calculi.^17^ In this study, transurethral ureteroscopy lithotripsy, minimally-invasive percutaneous nephrolithotomy and retroperitoneal laparoscopic ureterolithotomy were compared to provide a reference for the clinical development of therapeutic strategies.

In summary, the physical conditions of patients should be given first priority in treatment method selection. Meanwhile, surgeons should do their best to alleviate suffering symptoms timely and to reduce economic burden. Obviously, there are both advantages and disadvantages for the three surgical protocols in treating impacted upper ureteral calculi. In clinic, surgeons should comprehensively assess the specific clinical conditions of patients to find out the optimum one to improve clinical outcomes and to ensure surgery safety.

## Authors Contributions:

**LYH** **:** Study design. **ZZY: **Manuscript preparation. **XA and DHT: **Data collection.

**GLJ:** Data analysis. **ZJ: **Manuscript revision and data analysis.

## References

[1] Zheng B, Zhan HJ, Chen Y (2011). Comparison of three minimally invasive surgical procedures for 386 cases of upper ureteral calculi with un-severe hydronephrosis. Chongqing Med J.

[2] Djian MC, Blanchet B, Pesce F, Sermet A, Disdet M, Vazquez V (2012). Comparison of the time to extubation after use of remifentanil or sufentanil in combination with propofol as anesthesia in adults undergoing nonemergency. Clin Ther.

[3] Li B, Zhao QL, Li Q (2010). Minimally invasive treatment of ureteral calculi. Shandong Med J.

[4] Chen XF (2010). Interpretation of guidelines on urolithiasis. J Modern Urol.

[5] Liu ZH, Zhou XF (2010). Treatment progress of lower ureteral calculi. Chinese J Endourology (Electronic Version).

[6] Bauer C, Hentz JG, Ducrocq X, Meyer N, Oswald-Mammosser M, Steib A (2012). Lung Function after lobectomy: A randomized, double-blinded trial comparing thoracic epidural ropivacaine/sufentanil and intravenous morphine for patient-controlled analgesia. Anesth Analg.

[7] Liu GH, Wu SD, Gu NQ, He XP (2012). Ureteroscopy pneumatic lithotripsy for 38 cases of ureteral calculi and severe hydronephrosis. J Guangxi Med Uni.

[8] Wu CF, Chen CS, Lin WY, Shee JJ, Lin CL, Chen Y (2010). Threapeutic options for proximal ureter stone: extracorporeal shock wave lithotripsy vs semirigid extracorporeal with holmium. Urology.

[9] Lai GP, Chen Y, Pan WB, Liang C, Long H (2011). Comparison between the therapeutic effects of minimally invasive percutaneous nephrolithotomy and transurethral ureteroscope lithotripsy on impacted upper ureteral calculi after lithotripsy failure. J Difficult Complicated Cases.

[10] Goel A, Hemal AK (2013). Evaluation of role of retroperitoneoscopic pyelolithotomy and its comparison with percutaneous nephrolithotripsy. Int Urol Nephrol.

[11] Wang J, Liu GL, Yi R (2010). Comparison between the therapeutic effects of minimally invasive percutaneous nephrolithotomy and transurethral ureteroscope lithotripsy on impacted upper ureteral calculi. J Nanchang Uni Med Sci.

[12] Rofeim O, Yohannes P, Badlani GH (2011). Does laparoscopic ureterolithotomy replace shock-wave lithotripsy or ureteroscopy for ureteral stones. Curr Opin Urol.

[13] Li BJ (2012). Progression of minimally invasive therapy for upper ureteral calculi. Chinese J New Clin Med.

[14] Barakat TS, El-Nahas AR, Shoma AM, Shokeir AA (2013). Ureteroscopy for Upper Ureteral Stones: Overcoming the Difficulties of the Rigid Approach. Difficult Cases Endourol.

[15] Gao P, Zhu J, Zhou Y, Shan Y (2013). Full-length ureteral avulsion caused by ureteroscopy: report of one case cured by pyeloureterostomy, greater omentum investment, and ureterovesical anastomosis. Urolithiasis.

[16] Cosentino M, Palou J, Gaya JM, Breda A, Rodriguez-Faba O, Villavicencio-Mavrich H (2013). Upper urinary tract urothelial cell carcinoma: location as a predictive factor for concomitant bladder carcinoma. World J Urol.

[17] Yang Z, Song L, Xie D, Hu M, Peng Z, Liu T (2012). Comparative Study of Outcome in Treating Upper Ureteral Impacted Stones Using Minimally Invasive Percutaneous Nephrolithotomy With Aid of Patented System or Transurethral Ureteroscopy. Urology.

